# Supplementary figures and images for: Predicting Meridian in Chinese traditional medicine using machine learning approaches
Source: PLoS Comput Biol. 2019 Nov 25;15(11):e1007249. doi: 10.1371/journal.pcbi.1007249 (PMC6876772; doi:10.1371/journal.pcbi.1007249)

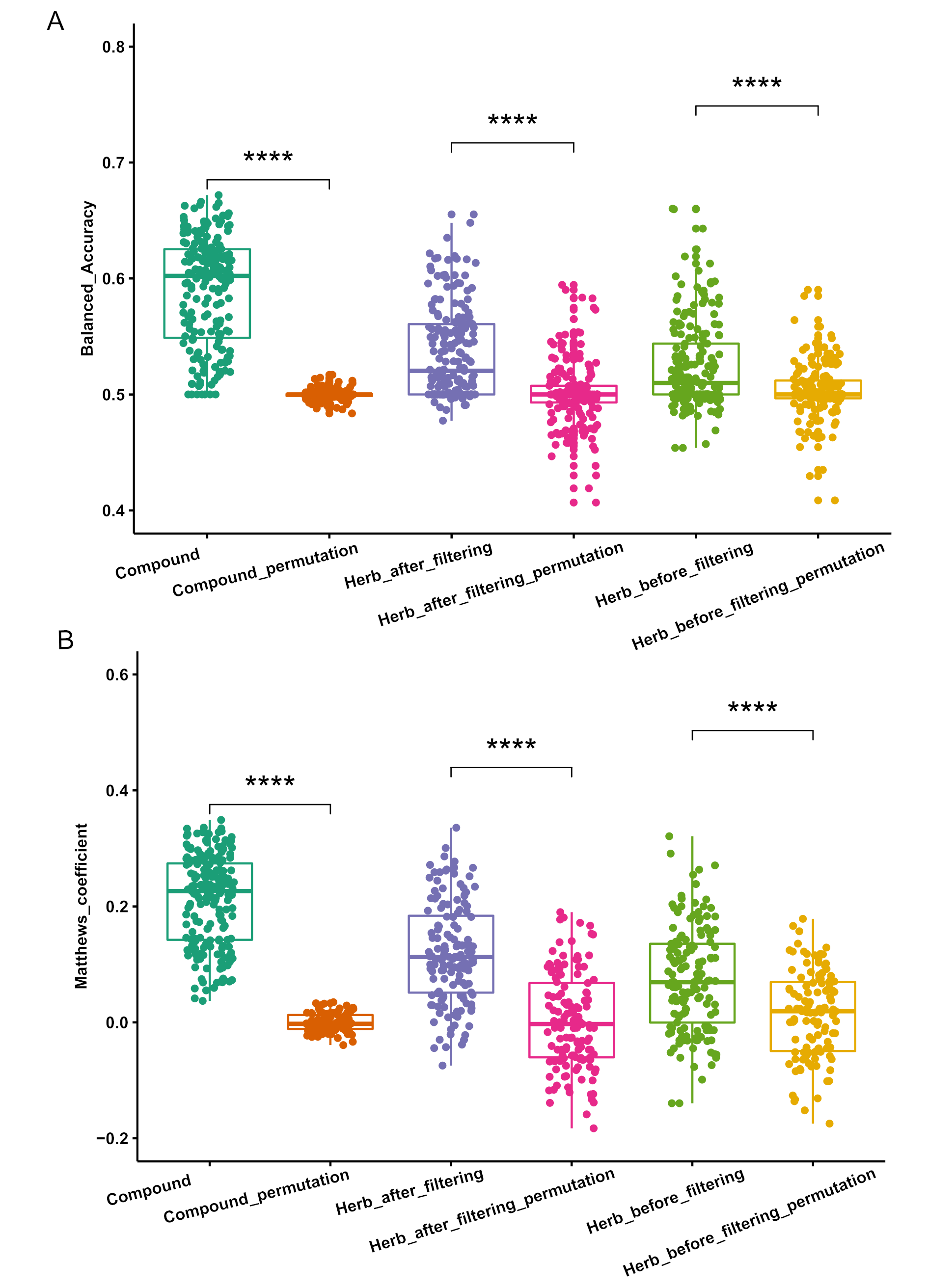

Supplement: S1 Fig — Balanced Accuracy (A) and Matthews correlation (B) for all the machine learning methods on the real data as compared to permutated data at the compound and herb levels. ****: p-value < 0.0001. (TIF) [file pcbi.1007249.s001.tif]

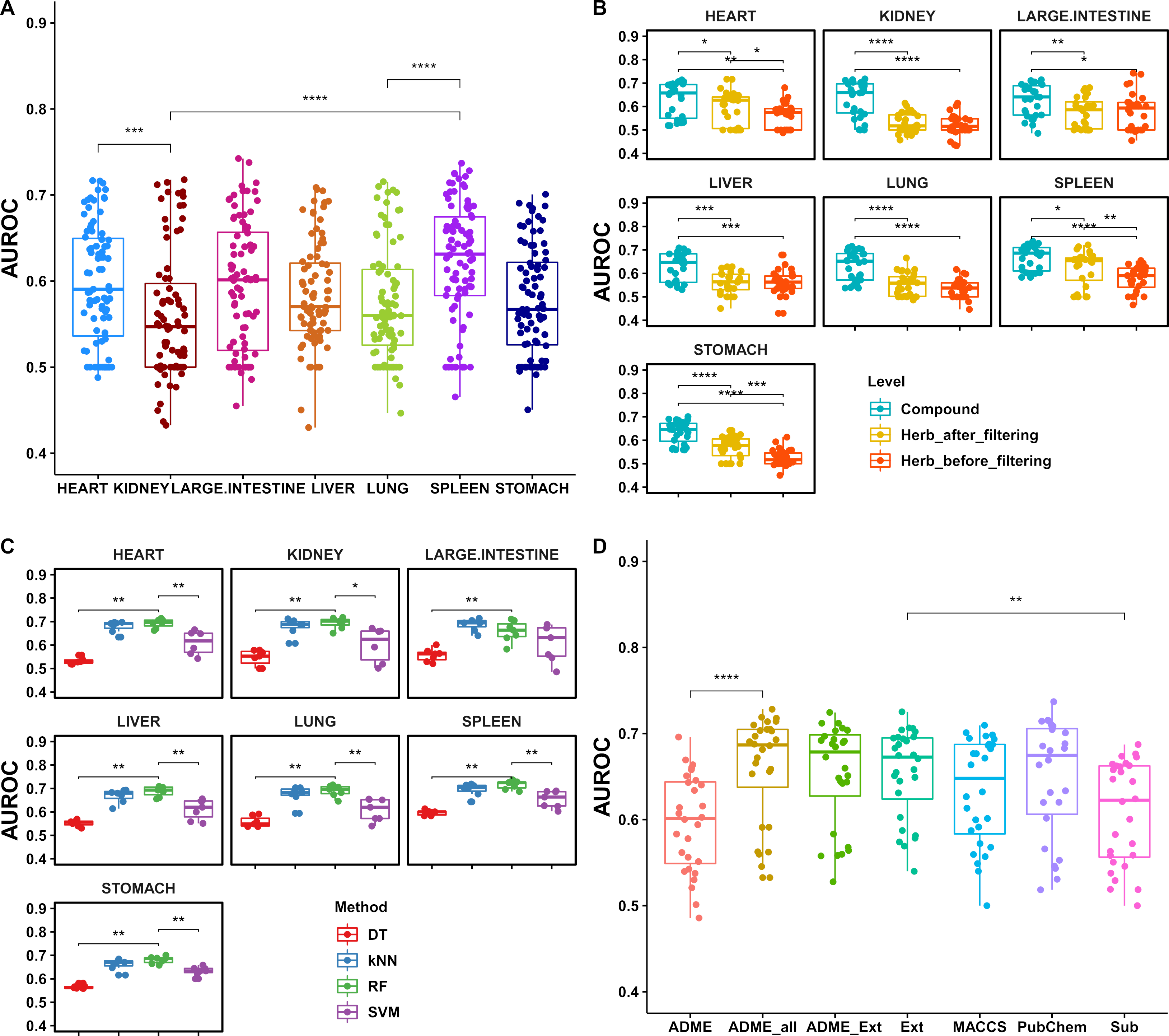

Supplement: S2 Fig — (A) The overall AUROC for the seven Meridians. (B) The AUROC at the three data levels (compound-level, herb-level before and after ADME filtering). (C) The AUROC for the five machine learning methods at the compound level. (D) The AUROC for the ADME and fingerprint feature types at the compound level. Wilcox rank sum test. *: p < 0.05; **: p < 0.01; ***: p < 0.001; ****: p < 0.0001 (TIF) [file pcbi.1007249.s002.tif]

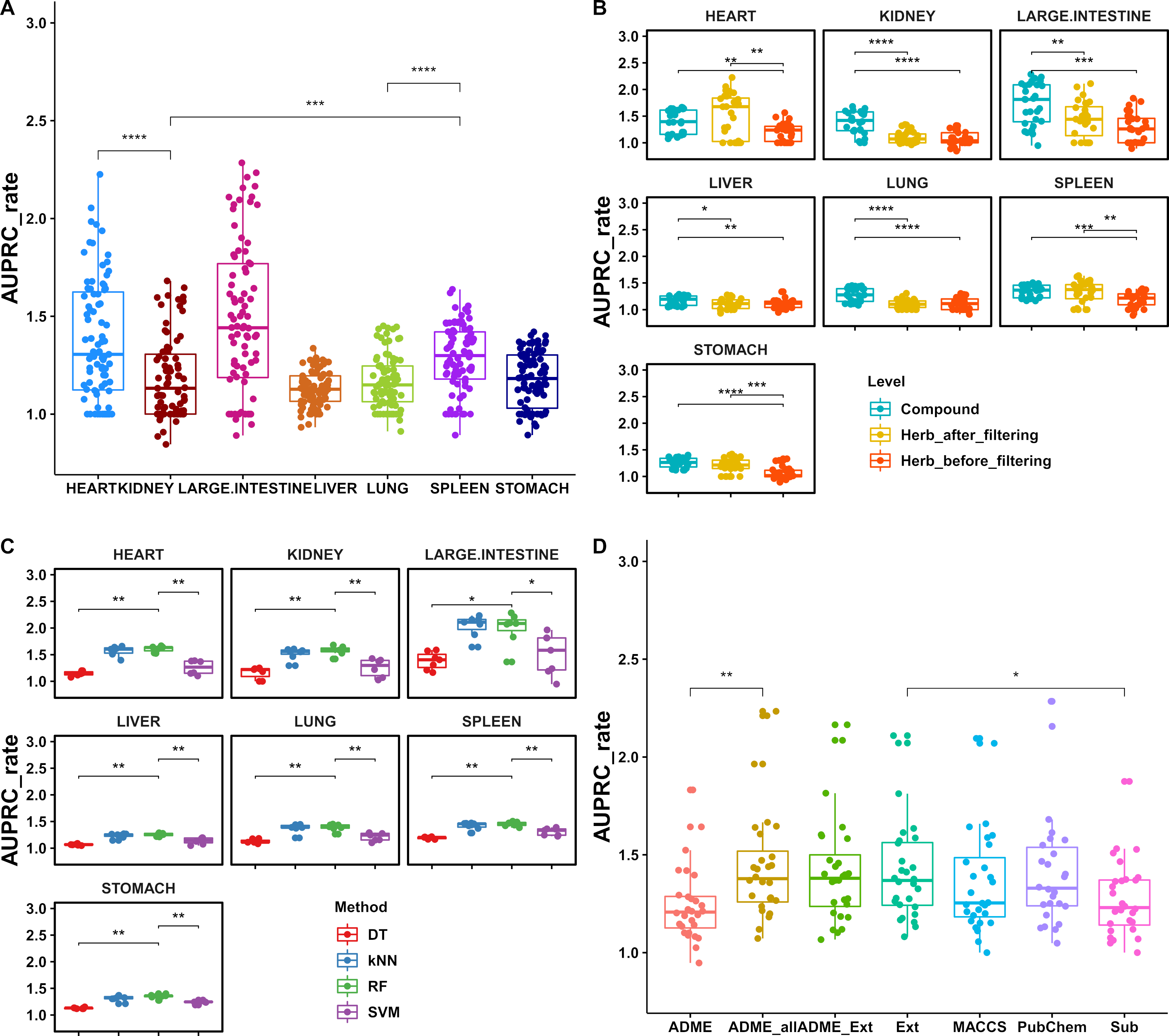

Supplement: S3 Fig — (A) The overall AUPRC ratio for the seven Meridians. (B) The AUPRC ratio at the three data levels (compound-level, herb-level before and after ADME filtering). (C) The AUPRC ratio for the five machine learning methods at the compound level. (D) The AUPRC ratio for the ADME and fingerprint feature types at the compound level. Wilcox rank sum test. *: p < 0.05; **: p < 0.01; ***: p < 0.001; ****: p < 0.0001. (TIF) [file pcbi.1007249.s003.tif]

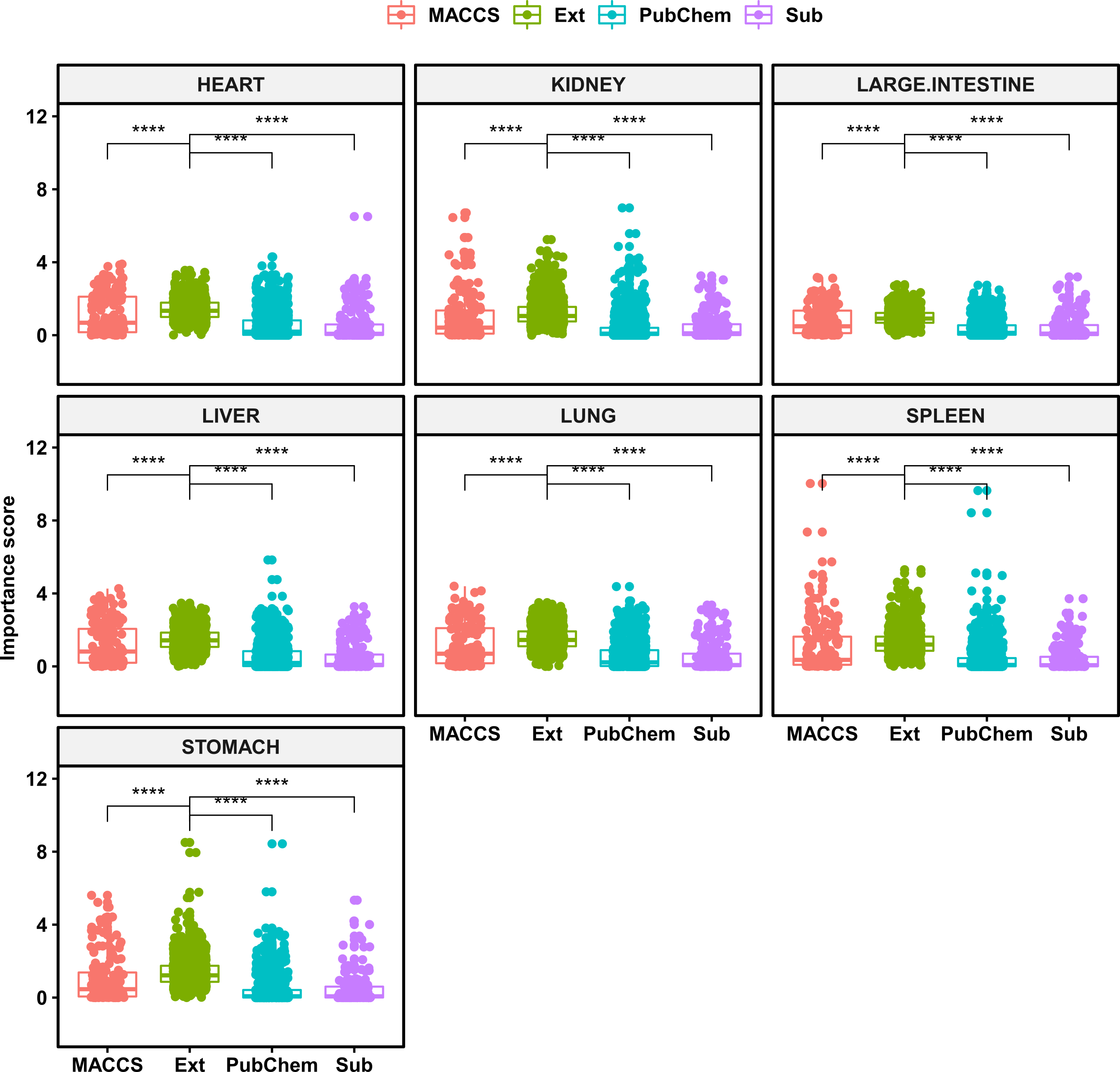

Supplement: S4 Fig — (TIF) [file pcbi.1007249.s004.tif]
