# Supplementary material for: Predicting Meridian in Chinese traditional medicine using machine learning approaches
Source: PLoS Comput Biol. 2019 Nov 25;15(11):e1007249. doi: 10.1371/journal.pcbi.1007249 (PMC6876772; doi:10.1371/journal.pcbi.1007249)
Supplement: Attachment — Submitted filename: PLoS_R1_response_letter_final_v2.docx [file pcbi.1007249.s011.docx]

**Reviewer #1:** This research presents a very interesting idea: using traditional machine learning algorithms and fingerprints of chemical compounds in herbs to predict herbs' Meridians. The manuscript is well organized and clearly written, and a large amount of computation is performed.

**Response:** We thank the Reviewer for the overall positive comments. Please find our point-by-point response below.

1. First, since an herb could belong to multiple Meridians as clearly stated by authors, this is a typical multiclass classification problem. However, this is totally ignored by the authors. From the method descriptions, they seem to use one-vs-rest approach. The authors need to explain why one-vs-rest is a good approach for this multiclass classification problem.

**Response:** Multi-label classification is the problem of classifying instances into one or more classes at the same time [1], while multi-class classification is the problem of classifying instances into one of three or more mutually exclusive classes [2]. We can treat both Multi-class classification and Multi-label classification as multiple binary classification problems [3]. In detail, the strategy is training a single classifier per class, with the samples of that class as positive samples and all other samples as negatives. In evaluation process, Multi-class classification uses the base classifiers to produce a real-valued confidence score for its decision as one sample only belongs to one of those classifiers. In contrast, Multi-label classification predicts the result of each label independent of each other. In TCM, an herb usually is classified into two or more Meridians at the same time (**Fig 2A-B** in the manuscript, also shown below). Therefore, we considered the prediction of Meridians rather a Multi-label classification problem.

**Fig 2.** Herb-Meridian and Compound-Meridian distributions. (A-B) The colour bars at the bottom left represent the frequency of herbs or compounds for each of the seven major Meridians, which can be further collapsed into subclasses depending on whether an herb or a compound is shared by one or several Meridians. The vertical bars show the frequency of herbs or compounds for a particular subclass of Meridian combination, as indicated by the connected lines below the x-axis between the Meridians. (C-D) The Jaccard coefficients between the Meridian pairs at the herb and the compound levels. The size of blue circles on the upper diagonal shows the degree of the similarity.

For the Meridian prediction problem, however, we found that the Jaccard indexes between the Meridians are generally low (maximally 0.31 at the herb level and maximally 0.42 at the compound level), suggesting weak correlations between them (**Fig 2C-D**). Therefore, we decided to treat each Meridian as independent response variable and ran machine learning predictions using separate classifiers, i.e. the one-vs-rest strategy. The advantages of this strategy is that we can identify the top predictors for each Meridian. We have explained the rationale of using the one-vs-rest strategy in more details in the Discussion (Page 12).

As suggested by the Reviewer, we have also tried the strategy of predicting multiple Meridians simultaneously using artificial neural network. Please find the results and discussion in our Response to the next comment.

2. Second, relevant to the first issue, neural network models could be used for this multiclass classification problem without such one-vs-rest approach. Have the authors tried any NN models? Did NN models perform poorly in comparison with these traditional algorithms?

**Response:** Thanks for the suggestion. We have tried the multilayer perceptron (MLP) model which is a class of feedforward artificial neural network. The MLP model allows the prediction of multiple Meridians simultaneously. However, as shown in the **Figure X1** below, MLP performed generally worse compared to the top models (e.g. RF and kNN) across all the major seven Meridians.

**Fig X1.**The AUROC for the five machine learning approaches.

Therefore, we have decided not to report the results of MLP, as the main focus of the manuscript is to show that the Meridians of TCM can be predicted with herb and compound features. As a future step we will try to improve the prediction accuracy using more advanced machine learning and artificial intelligence methods. Meanwhile, we will also improve the quality of data by searching more comprehensively the literature about the actual components of TCM herbs and their bioactivities. We believe that our manuscript may provide the first evidence that bioinformatics and systems pharmacology approaches become feasible for interrogating the concepts of TCM, with all the available data that has been accumulated and curated. We have explained the rationale and limitation of our manuscript in more details in the Discussion (Pages 14-15).

The implementation code for the MLP analysis has been upload to GitHub at:

<https://github.com/herb-medicne/Meridian-prediction/blob/master/multi-label%20neural%20network%20model/NN_model_multi-label.py>

3. Third, apparently, the data is quite unbalanced. Machine learning models are generally very sensitive to "unbalancedness" of the training data. Authors did not discuss this at all. Correspondingly, numbers of positive and negative samples should be added in **"Supplementary Table 3.xlsx".**

**Response:** We totally agree with the comment. In fact, as described in Result section 2.1 (Page 6), five Meridians (Bladder, Gallbladder, Small Intestine, Cardiovascular and Three End) that were extremely unbalanced were discarded in the machine learning analyses. The remaining seven Meridians have sufficient number of positive cases, ranging from 111 (17.2%) to 333 (51.5%) out of total 646 herbs. At the compound level, the data imbalance problem is less severe, ranging from 20% to 57.4% positive labels (**Fig 2** and new **Table S2**). To minimize the sensitivity of machine learning models on the data imbalance, we have applied cross validation to choose the best model using the training data, and then evaluate its performance on the independent test data (Method section 4.5, Page 21). In addition, to have a more comprehensive comparison between the machine learning models, we have utilized multiple metrics to evaluate the model performance, including Accuracy, Balanced accuracy, Matthews correlation and, as suggested by the Reviewers, AUROC and AUPRC (Method section 4.5, Page 22). Balanced accuracy, Matthews correlation, AUPRC and AUPRC are common metrics that work sufficiently well with unbalanced data. For the sake of consistency, we chose the Balanced accuracy as the main metric to compare the machine learning algorithms.

We have discussed the consideration of data imbalance in our machine learning framework in more details in Method section 4.5 (Pages 21-22), and also have reported the number of positive and negative samples as a new **Table S2** (shown below).

**Table S4.** The numbers of positive and negative samples for each Meridian at the herb and the compound levels

| Level | Meridian | Positive | Negative |
| --- | --- | --- | --- |
| Herb | Liver | 333 | 313 |
| Herb | Lung | 237 | 409 |
| Herb | Kidney | 181 | 465 |
| Herb | Heart | 155 | 491 |
| Herb | Large Intestine | 111 | 535 |
| Herb | Spleen | 213 | 433 |
| Herb | Stomach | 235 | 411 |
| Herb_filtered | Liver | 291 | 292 |
| Herb_filtered | Lung | 218 | 365 |
| Herb_filtered | Kidney | 167 | 416 |
| Herb_filtered | Heart | 140 | 443 |
| Herb_filtered | Large Intestine | 98 | 485 |
| Herb_filtered | Spleen | 193 | 390 |
| Herb_filtered | Stomach | 219 | 364 |
| Compound_level | Liver | 5771 | 4282 |
| Compound_level | Lung | 4491 | 5562 |
| Compound_level | Kidney | 3702 | 6351 |
| Compound_level | Heart | 3742 | 6311 |
| Compound_level | Large Intestine | 2010 | 8043 |
| Compound_level | Spleen | 4423 | 5630 |
| Compound_level | Stomach | 4579 | 5474 |

4. Fourth, for unbalanced data, AUC-ROC (the area under the receiver operating characteristic curve) is a widely used metrics; I highly recommend AUC-ROC numbers are calculated.

**Response:** As suggested, we have included the AUC results of ROC in new **Fig S2** (shown below) as well as in **Supplementary Table 3**. The top models across the seven major Meridians can reach AUROC higher than 0.7, in line with the Balanced accuracy results.

**Fig S2.** Evaluation of the machine learning model predictions by AUROC (The area under the receiver operating characteristic curve). (A) The overall AUROC for the seven Meridians. (B) The AUROC at the three data levels (compound-level, herb-level before and after ADME filtering). (C) The AUROC for the five machine learning methods at the compound level. (D) The AUROC for the ADME and fingerprint feature types at the compound level. Wilcox rank sum test. *: p < 0.05; **: p < 0.01; ***: p < 0.001; ****: p < 0.000

**Reviewer #2:** Wang and colleagues proposed a comprehensive machine learning-based study to predict Meridian in Chinese Traditional Medicine. They integrated multiple types of molecular fingerprints and ADME properties of active compounds in herbs. They then evaluated four different machine learning algorithms by combing different types of fingerprints and ADME properties, which is quite a novel and comprehensive insight. Some machine learning models reveal good accuracy in predicting herb-Meridian associations in cross validation. This is an impressive study which offers powerful machine learning-based approaches for evaluations of Meridian by Chinese Traditional Medicine. The main findings are well presented and the manuscript is well written. Several specific comments may help improve the manuscript further.

**Response:** We thank the Reviewer for the overall positive comments. Please find our point-by-point response below.

1. The reviewers appreciated that the authors collected large-scale herbs with specific ingredients from database. However, each active ingredient has different concentration across herbs. The authors use equal weight (concentration) for each ingredient for calculation of molecular fingerprints and ADME properties to build machine learning models. This limitation has to be well explained or discussed in the revised manuscript.

**Response:** We totally agree on the limitation of our modeling strategies. In fact, such a limitation may explain why the machine learning models performed less accurately at the herb level as compared to the compound level. As discussed in Page 14, in order to determine the chemical fingerprint features for an herb, we took the average of its compound features, based on the assumption that all the ingredient compounds are equally contributing to the pharmacology of the herb. This was likely an oversimplification of the actual mechanisms of action for a majority of herbs. However, the biological roles about the ingredient compounds were largely missing from TCMID and other resources, suggesting that the actual contributions of these ingredient compounds have not been thoroughly resolved. We have highlighted the need for more quantitative information about the ingredient concentrations in the herbs in the Discussion at Pages 14-15.

2. The authors only evaluated accuracy only for machine learning models. Several comprehensive indexes, such as AUC (area under ROC), and precision-recall curves should be added.

**Response:** The AUC for ROC curves were calculated and reported in new **Fig S2** (shown below) and also in **Table S3**. The AUC for precision-recall curves (PRC) curves were calculated and reported in **Table S3.** The AUPRC ratio, defined as the actual AUPRC divided by the reference AUPRC of random prediction is also shown in new **Fig S3** (shown below)**.** For the sake of consistency, we chose the Balanced accuracy as the main metric to compare the machine learning algorithms. However, the main conclusion that machine learning algorithms can predict Meridians still holds.

**Fig S2.** Evaluation of the machine learning model predictions by AUROC (The area under the receiver operating characteristic curve). (A) The overall AUROC for the seven Meridians. (B) The AUROC at the three data levels (compound-level, herb-level before and after ADME filtering). (C) The AUROC for the five machine learning methods at the compound level. (D) The AUROC for the ADME and fingerprint feature types at the compound level. Wilcox rank sum test. *: p < 0.05; **: p < 0.01; ***: p < 0.001; ****: p < 0.0001

**Fig S3.** Evaluation of the machine learning model predictions by AUPRC ratio, defined as the actual AUPRC divided by the baseline of random prediction. (A) The overall AUPRC ratio for the seven Meridians. (B) The AUPRC ratio at the three data levels (compound-level, herb-level before and after ADME filtering). (C) The AUPRC ratio for the five machine learning methods at the compound level. (D) The AUPRC ratio for the ADME and fingerprint feature types at the compound level. Wilcox rank sum test. *: p < 0.05; **: p < 0.01; ***: p < 0.001; ****: p < 0.0001.

3. The authors integrated both molecular fingerprints and ADME properties for building models. However, the reviewer cannot find how they integrated ADME properties in **Fig 1**.

**Response:** We considered ADME properties as features of the herbs and compounds, similar as molecular fingerprints. Therefore, the legend of **Fig 1** including F1, F2, etc. represent both ADME features and fingerprint features. However, to simplify the work flow, we showed the values of features as binary. We have added the explanation of legends in **Fig 1**.

4. The authors systematically evaluated four different machine learning algorithms in this study. More details of parameters of machine learning models are suggested to be provided. For example, which k used for kNN, which function (kernel or linear) used for SVM, how many trees used for Random forest, etc. The authors may get better performance if they optimize tree number in random forest models.

**Response:** We chose the caret package in R to tune the parameters using the default setting. The caret package utilized resampling to optimize the parameters. Details of the parameters can be found in the new **Table S6** (shown below).

**Table S6**. Parameters of the machine learning models

| **Model** | **Method value in caret** | **Libraries** | **Tuning Parameters** |
| --- | --- | --- | --- |
| CART | rpart | rpart | cp = 0.03846154, 0.05769231, 0.07692308 |
| Random Forest | rf | randomForest | mtry = 2, 62, 1939 |
| k-Nearest Neighbors | knn |  | K = 5, 7, 9 |
| Support Vector Machines | svmLinear | kernlab | C, Linear Kernel |

5. The authors calculated ADME properties using public tools. One popular tool, admetSAR should be discussed.

**Response:** We have added the discussion about admetSAR at Page 13:

“More relevant factors that may affect the ADME of herb medicine are expected to enhance the model prediction results. For example, another popular tool called admetSAR has been recently updated, which can provide 47 models for a more comprehensive evaluation of ADME [69, 70].”

6. It is impressive that the authors found that RF model shows the best performance for large intestine as several key ADME properties are highly correlated with large intestine. Could the authors evaluate the performance of RF models on large intestine using ADME properties only.

**Response:** Based on the metric of accuracy, we showed that RF achieved the best performance for Large Intestine. However, as suggested, the accuracy metric may be overestimating the performance for unbalanced data. Therefore, we decided to stick to the Balanced accuracy as the major metric to evaluate machine learning predictions. Therefore, we have replaced the Accuracy with Balanced accuracy in **Fig 2** and **Table 2** (shown below).

**Fig 2.** Evaluation of the machine learning model predictions. (A) The overall Balanced accuracy for the seven Meridians. Dashed line indicated the level of 0.65. (B) The Balanced accuracy at the three data levels (compound-level, herb-level before and after ADME filtering). (C) The balanced accuracy for the four machine learning methods at the compound level. (D) The balanced accuracy for the ADME and fingerprint feature types at the compound level. Wilcox rank sum test. *: p < 0.05; **: p < 0.01; ***: p < 0.001; ****: p < 0.0001.

**Table 2**. The balanced accuracy that was achieved for each Meridian at the compound level by Random Forest using all the available features.

| Meridian | **Feature** | **Method** | **Balanced Accuracy** |
| --- | --- | --- | --- |
| Heart | ADME + All fingerprint | RF | 0.65 |
| Kidney | ADME + All fingerprint | RF | 0.65 |
| Large intestine | ADME + All fingerprint | RF | 0.62 |
| Liver | ADME + All fingerprint | RF | 0.65 |
| Lung | ADME + All fingerprint | RF | 0.64 |
| Spleen | ADME + All fingerprint | RF | 0.67 |
| Stomach | ADME + All fingerprint | RF | 0.65 |

Based on the new Table 2, the performance for each Meridian is rather comparable at the same level. Therefore, we may not conclude that RF model achieved the best performance for Large intestine.

However, according to the AUPRC ratio (new **Fig S3A**, shown in response to comment No.2), Large intestine achieved the best AUPRC. Of all the seven Meridians, Large intestine also achieved the best AUPRC if using only ADME (new **Table 3**, shown below). It seemed that indeed there is an evidence that Large intestine is the most predictable for positive cases, as ADME properties tend to be more relevant for this particular Meridian. However, we felt that such an observation might need more in-depth functional and pharmacological analysis that is beyond the scope of current work. We hope that in the future we can collaborate with TCM researchers to analyze more details of the connection between ADME and Large intestine Meridian. We have included the results in the discussions at Page 13.

**Table 3.** The AUPRC ratio that was achieved for each Meridian at the compound level by Random Forest using ADME feature only.

| **Meridian** | **Method** | **AUPRC ratio** |
| --- | --- | --- |
| Large intestine | RF | 2.29 |
| Heart | RF | 1.67 |
| Spleen | RF | 1.51 |
| Kidney | RF | 1.68 |
| Stomach | RF | 1.40 |
| Lung | RF | 1.45 |
| Liver | RF | 1.29 |

7. Several key refs related to polypharmacy (10.1038/s41467-019-09186-x) and polypharmacology of natural products (doi: 10.1093/bib/bbx045) should be discussed.

**Response:** Thanks for the suggestion. We have added these references in Introduction at Page 5:

“Cheng *et al.* proposed a network-based methodology that can identify clinically efficacious drug combinations for specific diseases, which might be potentially used to explain also the pharmacology of TCM herbs [26].”

“Fang *et al.* summarized various chemo-informatics, bioinformatics and systems biology resources for reconstructing drug–target networks of natural products [27].”
